# Hypothalamic amenorrhoea as an extraintestinal manifestation of Crohn's disease: A case report

**DOI:** 10.51866/cr.937

**Published:** 2025-10-01

**Authors:** Ibrahim Adibah, Omar Ahmad Akram, Nageshar Ashna, Vikiraman Tamileswari

**Affiliations:** 1 MD, MMed (O&G), Department of Obstetrics and Gynaecology, School of Medical Sciences, Universiti Sains Malaysia, Kubang Kerian, Kelantan, Malaysia. E-mail: dradibah@usm.my; 2 MD, MMed (O&G), Department of Obstetrics and Gynaecology, School of Medical Sciences, Universiti Sains Malaysia, Kubang Kerian, Kelantan, Malaysia.; 3 MBBS, Department of Obstetrics and Gynaecology, School of Medical Sciences, Universiti Sains Malaysia, Kubang Kerian, Kelantan, Malaysia.; 4 MD, Department of Obstetrics and Gynaecology, School of Medical Sciences, Universiti Sains Malaysia, Kubang Kerian, Kelantan, Malaysia.

**Keywords:** Crohn's disease, Amenorrhoea, Functional hypothalamic amenorrhoea, Malnutrition, Inflammatory bowel disease

## Abstract

Crohn’s disease (CD), a chronic inflammatory bowel condition, although rare in Malaysia, can present with atypical systemic features, including reproductive dysfUnction. We report the case of a 31-year-old woman referred for secondary amenorrhoea and presumed endometriosis, later diagnosed with CD following worsening gastrointestinal symptoms and significant weight loss. Hormonal evaluation revealed hypothalamic-pituitary axis suppression secondary to malnutrition and systemic inflammation. Treatment with corticosteroids and nutritional rehabilitation led to weight gain and resumption of menses within 3 months. This case highlights the importance of recognising functional hypothalamic amenorrhoea as a reversible consequence of chronic illness and nutritional deficiency. It also underscores how systemic diseases such as CD may mimic gynaecological conditions, potentially delaying accurate diagnosis. Early multidisciplinary collaboration is essential in evaluating menstrual disturbances with overlapping gastrointestinal symptoms, especially in regions where inflammatory bowel disease remains uncommon.

## Introduction

Inflammatory bowel disease (IBD), namely ulcerative colitis (UC) and Crohn’s disease (CD), is relatively rare in Malaysia, with a mean incidence of 0.69 per 100,000 population. The rate is higher among Indians, at 1.91 per 100,000, and is followed by that among the Chinese and Malays, at 0.63 and 0.35, respectively.^1^ After 25 years of age, women have a 16%–47% higher risk of CD than men, according to an age-stratified meta-analysis.^2^

Patients with CD typically present with abdominal pain, chronic diarrhoea, weight loss and fatigue.^3^ In women, pain caused by CD might be mistaken as dysmenorrhoea, for which treatment may worsen the course of CD.^4,5^ CD can disrupt the hypothalamic-pituitary–ovarian axis through chronic inflammation, malnutrition and psychological stress, thus causing menstrual irregularities and amenorrhoea in 30% of cases,^6^ reduced fertility and adverse pregnancy outcomes.^7^

Due to the rarity of the disease in Asia, the abovementioned reproductive concerns are often under-recognised, especially when gastrointestinal symptoms are pronounced. This case report details the case of a young woman with newly diagnosed CD who was referred for evaluation of secondary amenorrhoea. Although she was initially suspected to have endometriosis, further workup revealed functional hypothalamic suppression, likely driven by chronic inflammation and significant weight loss due to CD. This case highlights the complex interplay between gastrointestinal and reproductive health in chronic disease. It also underscores the need for early recognition and intervention in evaluating menstrual disturbances, mainly when they occur in the setting of systemic symptoms.

## Case presentation

A 31-year-old Malay woman, para 1, whose last childbirth was 2 years ago, was referred to the gynaecology team for further investigation and management of secondary amenorrhoea. Her last menses occurred 6 months ago. She had attained her menarche at the age of 14 years with irregular cycles, once in 3 to 4 months, each lasting for 5 days. She experienced dysmenorrhoea, requiring occasional paracetamol or mefenamic acid ever since. She also reported passing out loose stools prior to or during menses.

About a year after her marriage, the patient conceived her child spontaneously. The pregnancy was uneventful. She delivered her child via spontaneous vaginal delivery at term. Her baby was alive and well.

The dysmenorrhoea worsened over the last year, causing her to have multiple hospital admissions. The pain improved with parenteral analgesia each time. Approximately 6 months prior to this presentation, during one of her admissions for dysmenorrhoea, a presumptive diagnosis of endometriosis was made based on her symptoms and clinical assessment. She was started on combined oral contraceptive pills (COCPs), which failed to improve her condition. Therefore, she did not return for any follow-up until her current admission.

Since the last 6 months, she reported persistent vomiting and diarrhoea, causing her to have poor oral intake and lethargy. She lost 20 kg over the same period. She stopped having menses since then. There was no history of tenesmus, dyschezia or haematochezia. Her family history was unremarkable.

**Figure 1:**
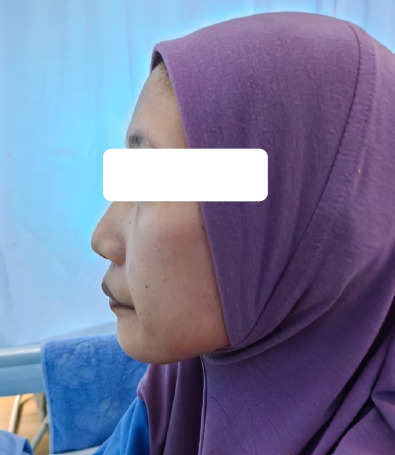
Temporal wasting indicating significant malnutrition.

**Figure 2:**
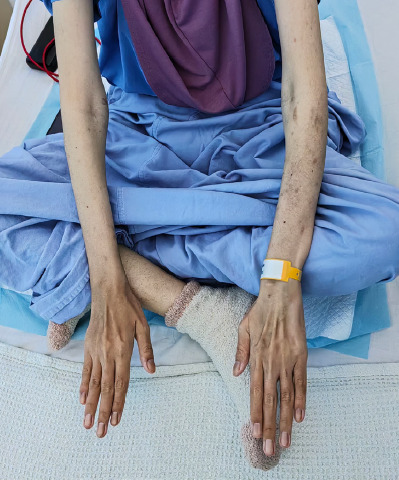
Severe muscle and fat wasting over the upper and lowerlimbs.

Upon assessment, she looked cachexic, with fat and muscle mass loss in the temporal region and upper and lower limbs (Figures 1 and 2). Her height was 162 cm, and her weight was 35 kg, yielding a BMI of 13.3 kg/m^2^. She was pale and dehydrated. Her vital signs were otherwise normal. Apart from having a scaphoid abdomen, the patient showed no other abnormality.

**Figure 3:**
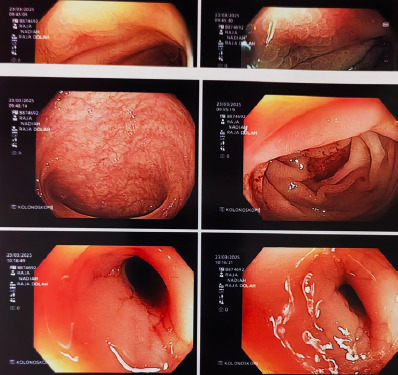
Colonoscopic image showing characteristic cobblestone appearance and ulcerationtypical ofCrohn’s disease.

Pelvic ultrasound revealed a normal-sized uterus with bilaterally normal ovaries. Colonoscopy demonstrated features of CD (Figure 3), which was confirmed by tissue histology. She showed markedly reduced follicle-stimulating hormone (0.43 IU/L), luteinising hormone (<0.3 IU/L) and oestradiol levels (<18.4 pmol/L), indicating hypothalamic-pituitary suppression.

The patient was treated with a course of corticosteroids, for which she gained1.5 kgafter only 2 days oftreatment. Shehad gained a total of 10 kg after 3 months of treatment and had spontaneouslyresumedhermenses.

## Discussion

This case illustrates the under-recognised reproductive consequences of CD in women of reproductive age, particularly in populations where CD remains uncommon. The patient presented with classic gastrointestinal symptoms of CD, yet her referral to the gynaecology team for secondary amenorrhoea prompted a broader multidisciplinary evaluation, leading to accurate diagnosis and appropriate management.

Menstrual dysfunction, including amenorrhoea, has been reported in up to 30% of women with IBD, particularly during active disease phases.^8,9^ In this patient, the profound weight loss, malnutrition and systemic inflammation were consistent with functional hypothalamic amenorrhoea. This condition arises from suppression of the hypothalamic-pituitary-gonadal axis due to inadequate energy availability, psychological stress and inflammatory cytokines.^9,10^

Malnutrition, especially a low BMI (<18.5 kg/m^2^), is a well-documented trigger for hypothalamic suppression, with leptin levels playing a central role. Leptin, secreted by adipose tissue, is necessary for GnRH pulsatility; low levels disrupt gonadotropin release, causing anovulation and amenorrhoea. This patient’s BMI of 13.3 kg/m^2^ strongly supports this mechanism. Chronic inflammation in CD can also impair gonadotropin secretion through elevated levels of cytokines such as IL-1, IL-6 and TNF-α.^11^

The initial misdiagnosis of endometriosis is common, given that abdominal pain and dysmenorrhoea can overlap with gastrointestinal symptoms. Studies have shown that women with CD frequently report dysmenorrhoea-like symptoms, which may be exacerbated during menstruation.^11^ However, treating such patients with hormonal therapies, COCPs in this patient, without addressing the underlying systemic illness, may mask key features and delay appropriate care. COCPs are believed to exacerbate the development and relapse of CD and UC.^12,13^ COCP users have a 46% higher risk of having CD or CD relapse (RR=1.46, 95% CI= 1.26-1.70).^14^ The exact mechanisms through which COCPs exacerbate CD are not known. However, oestrogen is linked to the inhibition of TH1-mediated cytokines and stimulation of TH2-mediated cytokines, which induce inflammatory conditions, finally leading to intestinal microvasculature thrombosis,^15-17^ believed to be the pathogenesis of IBD.^18,19^ Although patients with IBD generally maintain fertility during disease remission, active disease can impair fertility indirectly through complications such as malnutrition, pelvic inflammation and altered sexual behaviour due to pain or fatigue.^20^

Importantly, this case demonstrates that hypothalamic amenorrhoea is reversible with appropriate nutritional rehabilitation and control of the underlying disease. The patient’s rapid weight gain following corticosteroid therapy and the resumption of spontaneous menses support this outcome. This reinforces the need for early identification and multidisciplinary management involving gastroenterologists, gynaecologists and dietitians to optimise outcomes in similar cases.

In the Malaysian context, where CD remains rare and underdiagnosed, awareness of its systemic manifestations, including menstrual disturbances, is critical for timely diagnosis and holistic care. As the incidence of IBD increases in Asia, primary care physicians and specialists alike must maintain a high index of suspicion when evaluating women presenting with overlapping gastrointestinal and reproductive symptoms.

## Conclusion

- CD can present with reproductive symptoms such as secondary amenorrhoea, especially when complicated by malnutrition and systemic inflammation.
- Functional hypothalamic amenorrhoea should be considered in women with low BMI and menstrual irregularities.
- Multidisciplinary evaluation is key to avoiding misdiagnosis and guiding appropriate treatment.
- Early identification and treatment of underlying disease can reverse hypothalamic suppression and restore normal menstrual cycles.
- Greater awareness of the extraintestinal manifestations of CD, especially in regions with rising incidence, is crucial for holistic patient care.
